# SMaRT modulation of tau isoforms rescues cognitive and motor impairments in a preclinical model of tauopathy

**DOI:** 10.3389/fbioe.2022.951384

**Published:** 2022-10-05

**Authors:** Javier Andrés Muñiz, Carolina Lucía Facal, Leandro Urrutia, Ramiro Clerici-Delville, Ana Damianich, Juan E. Ferrario, Germán Falasco, María Elena Avale

**Affiliations:** ^1^ Consejo Nacional de Investigaciones Científicas y Técnicas, Instituto de Investigaciones en Ingeniería Genética y Biología Molecular “Dr Héctor N Torres”, Buenos Aires, Argentina; ^2^ Laboratorio De Imágenes Preclínicas, Centro de Imágenes Moleculares, FLENI, Buenos Aires, Argentina; ^3^ Universidad de Buenos Aires, Facultad de Ciencias Exactas y Naturales, Instituto de Biociencias, Biotecnología y Biología traslacional (iB3), Buenos Aires, Argentina

**Keywords:** MAPT, RNA therapy, Trans-Splicing, Dementia, FDG-microPET

## Abstract

Tau is a microtubule-associated protein predominantly expressed in neurons, which participates in microtubule polymerization and axonal transport. Abnormal tau metabolism leads to neurodegenerative diseases named tauopathies, such as Alzheimer’s disease and frontotemporal dementia. The alternative splicing of exon 10 (E10) in the primary transcript produces tau protein isoforms with three (3R) or four (4R) microtubule binding repeats, which are found in equal amounts in the normal adult human brain. Several tauopathies are associated with abnormal E10 alternative splicing, leading to an imbalance between 3R and 4R isoforms, which underlies disease. Correction of such imbalance represents a potential disease-modifying therapy for those tauopathies. We have previously optimized a *trans*-splicing RNA reprogramming strategy to modulate the 3R:4R tau content in a mouse model of tauopathy related to tau *mis*-splicing (htau mice), and showed that local modulation of E10 inclusion in the prefrontal cortex prevents cognitive decline, neuronal firing impairments and hyperphosphorylated tau accumulation. Furthermore, local shifting of 3R–4R tau into the striatum of htau mice prevented motor coordination deficits. However, a major bottleneck of our previous work is that local splicing regulation was performed in young mice, *before* the onset of pathological phenotypes. Here we tested whether regulation of tau E10 splicing could rescue tau pathology phenotypes in htau mice, *after* the onset of cognitive and motor impairments, comparable to early stages of human tauopathies. To determine phenotypic time course and affected brain nuclei, we assessed htau mice using behavioural tests and microPET FDG imaging over time, similarly to diagnosis methods used in patients. Based on these analyses, we performed local delivery of pre-*trans* splicing molecules to regulate E10 inclusion either into the medial prefrontal cortex (mPFC) or the striatum at 6-month-old once behavioral phenotypes and metabolic changes were detected. Tau isoforms modulation into the mPFC restored cognitive performance in mice that previously showed mild to severe memory impairment while motor coordination deficit was rescued after striatal injection of *trans*-splicing molecules. Our data suggest that tau regulation could recover pathological phenotypes early after phenotypic onset, raising promising perspectives for the use of RNA based therapies in tauopathies related to *MAPT* abnormal splicing.

## 1 Introduction

Tauopathies are a large group of neurodegenerative diseases, including Alzheimer’s disease (AD), frontotemporal dementia, progressive supranuclear palsy and Pick’s disease (Spillantini and Goedert, 2013). Current treatment is only symptomatic and there is no effective therapy to stop or slow the progression of the degenerative process. Tauopathies are characterised by intracellular accumulation of hyperphosphorylated Tau in insoluble neurofibrillary tangles (NFTs) (Grundke-Iqbal et al., 1986). Although NFTs are a hallmark of tauopathies and have been largely pointed as a causal of disease (Spires-Jones et al., 2011), it is also noteworthy that the lack of normal tau function might also underlie neuronal dysfunction, with the consequent manifestation of clinical signs of dementia (Avila, 2009; Medina et al., 2016; Catarina Silva and Haggarty, 2020).

Tau is normally expressed in neurons, highly enriched in axons, and is involved in a myriad of neuronal functions, including the regulation of microtubule dynamics and axonal transport, the formation of neuronal processes, and synaptic regulatory mechanisms (Morris et al., 2011). Tau is mainly bound to microtubules, through three or four microtubule binding domains (MBD). Under certain conditions, either due to altered sequence, abnormal post-transcriptional or post-translational processing, tau detaches from microtubules and accumulates in cell bodies and neurites, where it begins the formation of oligomers that finally accumulate in NFTs and were associated with a pathological frame (Bodea et al., 2016).

Human tau is encoded by the *MAPT* gene, comprising 16 exons, which produce six different isoforms in the adult brain by alternative splicing of exons 2, 3 and 10 (Andreadis, 2005). The alternative splicing of Exon 10 (E10) gives rise to tau isoforms with three (3R) or four (4R) microtubule binding repeats, which are found in equal amounts in the normal adult human brain (Goedert et al., 1989; Andreadis et al., 1992). Imbalances in tau 3R:4R isoforms ratio are known as a disease-causative phenomenon (Hutton et al., 1998). So far, near 100 mutations have been found in the *MAPT* gene [reviewed in (Spillantini and Goedert, 2013; Qian and Liu, 2014)] with about one third of them affecting E10 splicing and consequently, the normal 3R:4R balance (Niblock and Gallo, 2012). In addition, other non-genetic factors might also affect *MAPT* alternative splicing (Rösler et al., 2019), yielding a plethora of causes for 3R:4R imbalance related to dementia. Yet, no successful treatment has become available for those tauopathies, but early correction of abnormal E10 splicing arise as a potential therapeutic strategy to preclude or delay the onset of disease.

We have previously shown that the SMaRT strategy -spliceosome mediated RNA *trans*-splicing- (Rodriguez-Martin et al., 2005) is a suitable tool to modulate E10 inclusion in endogenous *MAPT* transcript, both in mouse and human neurons in culture (Avale et al., 2013; Lacovich et al., 2017), and into the adult mouse brain (Espíndola et al., 2018; Damianich et al., 2021). To test SMaRT modulation of Tau *in vivo*, we used the htau mouse model (Andorfer et al., 2003) which expresses a full length wild-type human *MAPT* transgene in an endogenous *Mapt−/−* background. Given the presence of the full *MAPT* gene, with all its intronic and regulatory sequences, this mouse provides a unique platform to test molecular tools for validating *MAPT* splicing modulation *in vivo*. Furthermore, in the htau mouse brain the splicing processing of the human *MAPT* transcript favours the production of 3R tau in the adult brain. Thus, compared to the wild-type adult mouse brain that produces only 4R tau, the htau brain bears an aberrant content of tau isoforms. In our previous *in vivo* studies, we observed that early modulation of abnormal tau isoforms contents precludes phenotypic impairments in htau mice. Yet, such tau isoforms modulation was performed in young mice at 2-3 months old, i.e., *before* detection of pathological phenotypes. However, many preventive therapies, although useful experimentally, are poorly translated for human neurodegenerative diseases that require a treatment *afte*r the onset of clinical symptoms. Hence, we sought to investigate whether correction of tau isoforms contents at more advanced stages of disease could also yield a phenotypic rescue. To this end, we analysed the time course of behavioural phenotypes and performed *in vivo* microPET imaging analyses in the hau model, to determine the early stages of disease, similarly to which is performed in patients. Then, we performed local tau splicing modulation into the medial prefrontal cortex (mPFC) or the striata of 6-month-old mice and detected significant improvements of cognitive and motor impairments.

## 2 Methods

### 2.1 Mice

All animal procedures were designed in accordance with the NIH Guidelines for the Care and Use of Laboratory Animals. Protocols were approved by ICAUC of INGEBI-CONICET and University of Buenos Aires. Mice were housed in standard conditions under 12 h dark/light cycle with *ad libitum* access to food and water. Htau transgenic mice, in a C57BL/6 background, were obtained from Jackson Laboratories (Bar Harbour, Maine, United States; B6.Cg-Mapttm1 (EGFP) Klt Tg (MAPT)8cPdav/J. Stock number: 005491). To confirm the presence of the human MAPT transgene and the mouse Mapt −/− background, all mice used in this study were genotyped by PCR as previously described (Avale et al., 2013). Male and female adult mice were used to conduct all the experiments described in this study. No differences were observed among genders and data was pooled.

### 2.2 Behavioural tests

All mice tested were sibling cohorts aged 3, 6, 9 and 12 months, as indicated. Experiments were performed between 13:00 h and 17:00 h under dim illumination, in a separated behavioural room, where mice were transferred in advance. Behavioural experiments were analysed by ANY-maze (Stoelting Co.). All arenas and devices were cleaned between subjects to minimise odour cues.

#### 2.2.1 Open field

Activity boxes (Med Associates Inc.) coupled to a computer interface (Activity Monitor software, Med Associates Inc.) were used to assess horizontal and vertical activity. Mice were placed in the centre of the empty acrylic boxes (40 × 40 × 40 cm) and their trajectories were recorded for 30 min by disruption of infrared photobeams separated by 2.5 cm that cross the x–y plane at two z-levels to determine total distance travelled, time spent the centre (a virtual square of 10 cm^2^) and periphery of the arena.

#### 2.2.2 Rotarod

Motor coordination was assessed as previously described (Damianich et al., 2021) in a rotating mill (MEdPC) at fixed speed of 24 rpm for 180 s. Maximum time on the rod and total number of falls were used for analyses.

#### 2.2.3 Novel object recognition

The test was performed as previously described (Espíndola et al., 2018) with minor modifications. Mice were individually habituated to the empty chamber (30 × 23 × 25 cm) for 10 min. Then placed into the chamber with two identical objects for 10 min. Three hours later, mice were tested for 3 min in the same chamber with two objects in the same position, but one object replaced by a novel one with different shape, colour and form. Objects used were like the ones used by Leger et al. (Leger et al., 2013). Each object was randomly assigned as novel or familiar, for each mouse. Time spent exploring each object was recorded. Discrimination index was calculated as the time spent exploring the novel object related to the total time spent exploring both objects.

#### 2.2.4 Elevated plus maze

The test was performed as described previously (Avale et al., 2004). Briefly, mice were placed into the central area of the maze (70 cm elevated black cross-shaped Perspex apparatus; arms: 35 × 5 cm wide; central square: 5 × 5 cm, closed arms enclosed by vertical walls and open arms with unprotected edges). Time spent in the open and closed arms during a 3 min exploration period was recorded by an overhead camera. An entry was counted only if all four paws were inside the arm.

### 2.3 Positron emission tomography

*In vivo* brain activity was analysed using tracer fluorinated glucose analog (18F-FDG), which localises in metabolically active tissues and accumulates in an activity-dependent manner. Animals were starved during 4 h and then injected with 25 μCi/gr of 18F-FDG i. p. and left undisturbed in an individual temperature-controlled (29°C) cage for 30 min during radiopharmaceutical incorporation. Mice were then anaesthetised using a mixture of isoflurane and O_2_ (inhalation, 4.5% induction and 1.5% maintenance dose) and maintained in a warm table (35°C) during the acquisition. Images were acquired using a preclinical PET TriFoil Lab-PET 4 (3.75 cm axial length) with a dual layer of LYSO and GSO crystals, assembled in phoswich pairs. Signal read out is based on an APD-Detection (Avalanche PhotoDiode). Image reconstruction was performed on emission data through 3D-OSEM (ordered subset expectation maximisation) iterative reconstruction (30 iterations). All images were co-registered and normalised to a 18F-FDG template. The quantitative brain image of each mouse is normalized to the total cortex to avoid bias in the analysis. Intensity normalisation was considered as a regressor variable for each factor using all-brain mean scaling (ANCOVA). Results are shown using a color scale representing a statistical parametric comparison between the groups, using the *t*-test (*p* < 0.05).

### 2.4 Lentiviral vectors

The PTM4R RNA molecule targets the tau transcript by a 125 nt binding domain complementary to the 3′ end of intron 9, followed by a branch point and the 3′AG splice acceptor site, as previously described (Rodriguez-Martin et al., 2005). The coding sequence of the PTM consists of tau exons 10 to 13. Control vector (LV-PTM4R-ΔTSD) carries the same PTM but lacks the *trans*-splicing domain. Lentiviral vectors (LV) for delivering control vector or tau PTM4R to induce *trans*-splicing were previously described (Avale et al., 2013; Espíndola et al., 2018; Damianich et al., 2021).

### 2.5 Stereotaxic injections

Lentiviral vectors were delivered into specific brain structures as previously described (Espíndola et al., 2018; Bordone et al., 2021) with minor modifications. Briefly, mice (males and females) aged 6 months (weight 25–30 g) were anaesthetised with isoflurane 0.5–2% (Baxter) and placed into a stereotactic frame (Stoelting CO.). The skull was exposed and bregma was identified. A 10.0 μL Hamilton syringe coupled to a 36G stainless steel tube (Cooper needleworks, United Kingdom) was used to inject 1.5 μL of lentiviral suspension (0.6 × 107 TU/ml; 0.2 μL/min) per site of injection, following coordinates described in the mouse atlas (Paxinos and Franklin, 2013). LV suspension was infused bilaterally, at 2 sites into the mPFC, coordinates (in mm): AP = +2.3, L = ±0.5, DV = -1.8 and -2.2; and four sites for the striatum, coordinates in mm: AP = +1.2, L = ±1.5, DV = −3.8 and −3; AP = +0.2, L = ±2, DV = −4 and −3. Animals were kept at 37°C during the surgery until full recovery. Immediately after surgery, mice received analgesic Aplonal (1 mg/kg, s.c.), repeated 24 h later. Any animal showing signs of pain or discomfort after surgery was sacrificed following the end point protocol.

### 2.6 Detection of tau mRNA isoforms

Total RNA was extracted from dissected mPFC or striata using the RNeasy Lipid Tissue Kit (Qiagen). Reverse transcription was performed with the TaqMan RT kit (Applied Biosystems) with an equimolar ratio of oligo (dT) and random hexamers. Each reaction contained 0.5 μg of RNA in a total volume of 10 μL. Reverse transcription conditions were: 10 min at 25°C, 30 min at 48°C, and a final step of 5 min at 95°C. To perform the relative quantification of 4R and 3R mRNA isoforms by real time PCR, specific pairs of primers were used per each isoform, either spanning the exons E9-E10 or E9/11-E11 respectively. Primers sequences for 3R human tau isoform (forward: 5′- AGG​CGG​GAA​GGT​GCA​AAT​AG - 3′ and reverse: 5’ - TCC​TGG​TTT​ATG​ATG​GAT​GTT - 3′). Primers for 4R tau (forward: 5’ - TCCACTGAGAACCTGAAG - 3′ and reverse: 5’ - TATCCTTTGAGCCACACT - 3′). The housekeeping gene used was Cyclophilin B (forward: 5’ - TGG​AGA​TGA​ATC​TGT​AGG​ACG​A - 3′ and reverse: 5’ - GAA​GTC​TCC​ACC​CTG​GAT​CA - 3′). qPCR reactions were performed in triplicate with 40 ng of cDNA and 5 μL of Power SYBR^®^ Green PCR Master Mix (Applied Biosystems) in a final volume of 10 μL. The Applied Biosystems 7500 Real-Time PCR System was used under the following cycling conditions: after initial denaturation at 95°C (10 min), 40 cycles at 95°C (10 s), the primers specific annealing temperature was 58°C (30 s) and elongation at 72°C (45 s). Data was analyzed with the 7500 Software (Applied Biosystems) to obtain the ΔCT per sample. Values per each isoform were standardized with the Cyclophilin B reference gene.

### 2.7 Protein extraction and western blotting

The mPFC and striatum were dissected and homogenised with a buffer containing 40 mM Tris-HCl and 10% glycerol (pH 7.4), containing a proteases and phosphatases inhibitor cocktail (Thermo Scientific). Equal amounts of total protein (determined by Pierce BCA Protein Assay Kit, Thermo Scientific) were separated on 12% SDS-Polyacrylamide gels (prepared with Acrylamide and N,N′-Methylenebisacrylamide 30%) and transferred using a semi-dry transfer system to nitrocellulose membranes (BioRad). Membranes were blocked in 5% (w/v) non-fat dry milk (Sancor, Argentina), 0.05% v/v Tween 20 in TBS (milk/1xTBS-T) for 1 h. After blocking, membranes were incubated overnight at 4°C with primary antibodies in the same blocking solution. Primary antibodies used were directed either against 3R tau (1:2000 Antitau 3-repeat isoform RD3; mouse monoclonal, Millipore), 4R tau (1:500 Antitau 4-repeat isoform RD4; mouse monoclonal, Millipore), total tau (1:10000; rabbit polyclonal; Dako, Denmark), Phospho-Ser202 (CP13 monoclonal, 1:300; from Peter Davies) and Phospho-Ser396-Ser404 (PHF-1 monoclonal; 1:500; from Peter Davies), mouse β-actin (mouse monoclonal, 1:10000; abcam, United Kingdom). After washing 3 times in TBS containing 0.05% v/v Tween 20, blots were incubated with the appropriate secondary antibody either horse anti-mouse (1:2000, Cell Signalling) or goat anti-rabbit (1:2000, Cell Signalling) for 2 h at room temperature. Proteins were visualised using ECL reagent (Thermo Scientific) exposing membranes on the GenegnomeXRQ (Syngene). Optical density was quantified using ImageJ software (Rasband).

### 2.8 Statistical analyzes

Data were analyzed with Prism GraphPad software. When large samples were available and normal distribution was assumed the three experimental groups were analyzed by 1-way analysis of variance (ANOVA) followed by Tukey’s multiple comparisons post-hoc tests as the software recommends; and the four experimental groups were analyzed by 2-way analysis of variance (ANOVA) followed by Sidak’s multiple comparisons post-hoc tests. When indicated, paired comparisons were performed by two-tailed student’s *t* test.

## 3 Results

### 3.1 Time course of cognitive and motor deficits in the htau model of tauopathy

**FIGURE 1:**
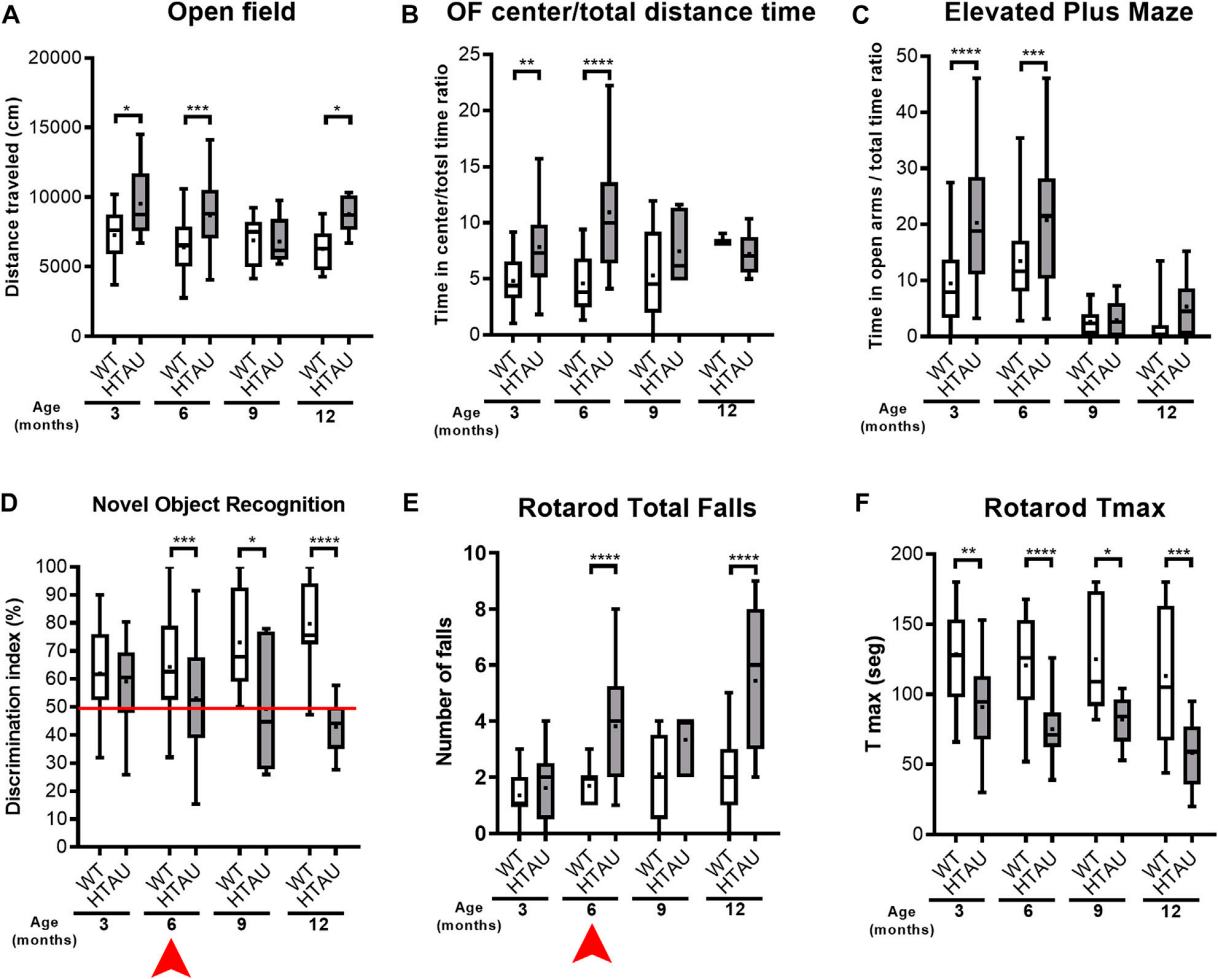
Time course of cognitive and motor deficits in htau mice. Htau and WT mice were tested in a battery of behavioural tests at 3, 6, 9 and 12 months of age. **(A)** Free exploration during 30 min in the open field indicates increased distance travelled in htau mice compared to WT (2-way ANOVA, followed by Sidak’s multiple comparisons test, *p < 0.05, ***p < 0.001, 3 months: WT *n* = 17, htau *n* = 15; 6 months: WT *n* = 31, htau *n* = 33, 9 months: WT *n* = 10, htau *n* = 7, 12 months: WT *n* = 14, htau *n* = 10). **(B)** Time spent in the centre of the open field arena was increased in htau mice at 3 and 6 months old (2-way ANOVA, followed by Sidak’s multiple comparisons test, **p < 0.01, ****p < 0.0001, 3 months: WT *n* = 17, htau *n* = 15; 6 months: WT *n* = 31, htau *n* = 33, 9 months: WT *n* = 10, htau *n* = 7, 12 months: WT *n* = 14, htau *n* = 10). **(C)** Elevated Plus Maze; htau mice showed increased preference to visit open arms at 3 and 6 months old (2-way ANOVA, followed by Sidak’s multiple comparisons test, ***p < 0.001, ****p < 0.0001, 3 months: WT *n* = 39, htau *n* = 42; 6 months: WT *n* = 60, htau *n* = 60, 9 months: WT *n* = 10, htau *n* = 7, 12 months: WT *n* = 17, htau *n* = 10). **(D)** Discrimination index (DI) in the Novel Object Recognition test associated with cognitive decline at 6 months-old until 12-month-old mice (2-way ANOVA, followed by Sidak’s multiple comparisons test, *p < 0.05, ***p < 0.001, ****p < 0.0001, 3 months: WT *n* = 39, htau *n* = 42; 6 months: WT *n* = 79, htau *n* = 76, 9 months: WT *n* = 10, htau *n* = 7, 12 months: WT *n* = 14, htau *n* = 18). **(E)** Number of falls in the rotarod test increased in htau mice from 6 months old (2-way-ANOVA, followed by Sidak’s multiple comparisons test, ****p < 0.0001, 3 months: WT *n* = 17, htau *n* = 15; 6 months: WT *n* = 23, htau *n* = 22, 9 months: WT *n* = 10, htau *n* = 7, 12 months: WT *n* = 16, htau *n* = 9). **(F)** Maximum time spent on to the rotating treadmill, indicating motor decline in htau mice from 3 months old (2-way-ANOVA, followed by Sidak’s multiple comparisons test, *p < 0.05, **p < 0.01, ***p < 0.001, ****p < 0.0001, ****p < 0.0001, 3 months: WT *n* = 17, htau *n* = 15; 6 months: WT *n* = 23, htau *n* = 22, 9 months: WT *n* = 10, htau *n* = 7, 12 months: WT *n* = 16, htau *n* = 9) Arrows indicate onset of phenotypic differences in htau mice.

To analyse behavioural phenotypic onset in the htau model, mice were tested in a battery of behavioural tasks at 3, 6, 9 and 12 months-old. When compared to WT controls, htau mice showed hyperlocomotion in the open field from 3 months old, while time spent in the centre of the arena was increased only at 3 and 6 months of age (Figures 1A,B). Moreover, in the elevated plus maze, young (3 to 6-month-old) but not old (9–12-month-old) mice showed preference to visit open arms (Figure 1C). Together these results suggest a reduction of risk avoidance behaviour in young htau mice which wears off in older mice.

We next analysed cognitive performance using the Novel Object Recognition (NOR) task (Figure 1D). A discrimination index (DI) above 50% reflects preference for the novel object while a score of 50% indicates no difference in exploring both objects, suggesting lack of memory for the familiar object. At 3 months old, DI was similar between htau and WT mice (60-70%), while 6-month-old htau mice showed no preference for the novel object, which persisted at 12 months, suggesting that the onset of cognitive impairment occurs between 3 and 6 months, consistent with previous reports (Polydoro et al., 2009; Espíndola et al., 2018).

Based on our previous findings of motor deficits in aged htau mice (Damianich et al., 2021), we assessed time course of motor coordination performance in the rotarod test. At 3 months-old, htau mice did not display a significant difference in total number of falls compared to WT controls (Figure 1E), but showed a mild impairment in the maximum time spent on the rod (TMAX) (Figure 1F). However, from 6 months old, motor coordination of htau significantly decreases compared to WT mice, both increasing the number falls (Figure 1E) and decreasing the time spent on the rod (Figure 1F). This deficit worsens between 9 and 12 months old. Taken together, behavioural analyses indicate that both motor and cognitive impairments of htau mice are already evident at 6 months, showing a progressive decline with ageing.

### 3.2 Time course changes in brain glucose uptake *in vivo* in htau mice

**FIGURE 2:**
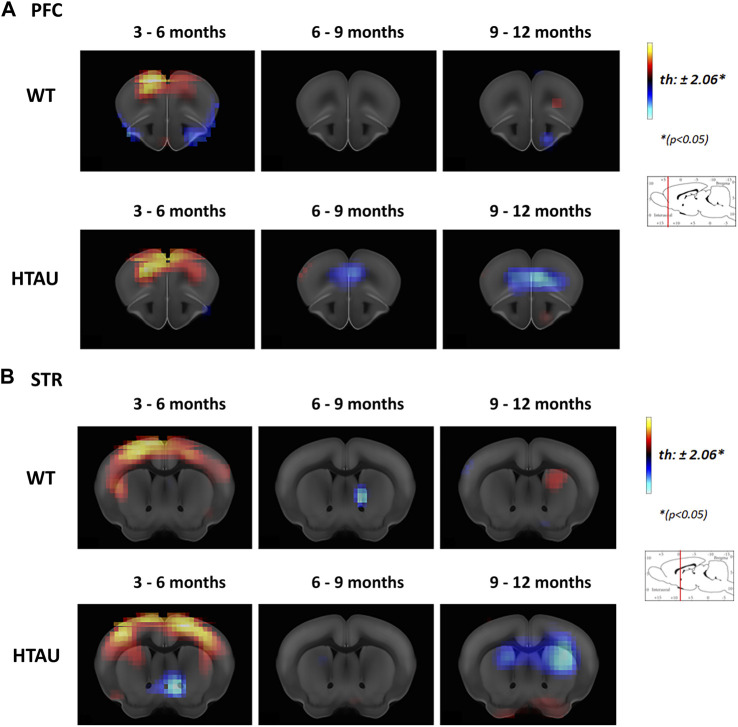
Age-related changes in brain metabolism in htau mice. *In vivo* brain glucose uptake was analysed by [18F]-FDG preclinical PET imaging in htau and WT mice at 3-, 6-, 9- and 12-month-old. The result is shown as a statistical parametric comparison between the groups. Color changes represent a statistical change, determined by *t-test* comparisons (*p < 0.05) between the groups compared (indicated in each panel). Red-yellow look up table side indicates [18F]-FDG uptake increases, while blue indicates uptake decrease. Coronal sections showing [18F]-FDG uptake changes in the Prefrontal cortex **(A)** and striatum **(B)** of htau and WT mice, between 3 and 6, 6 to 9 and 6–12 months old (WT *n* = 11; htau *n* = 9). Images shown represent T-maps of brain metabolism compared to former recorded activity of the same group.

**FIGURE 3:**
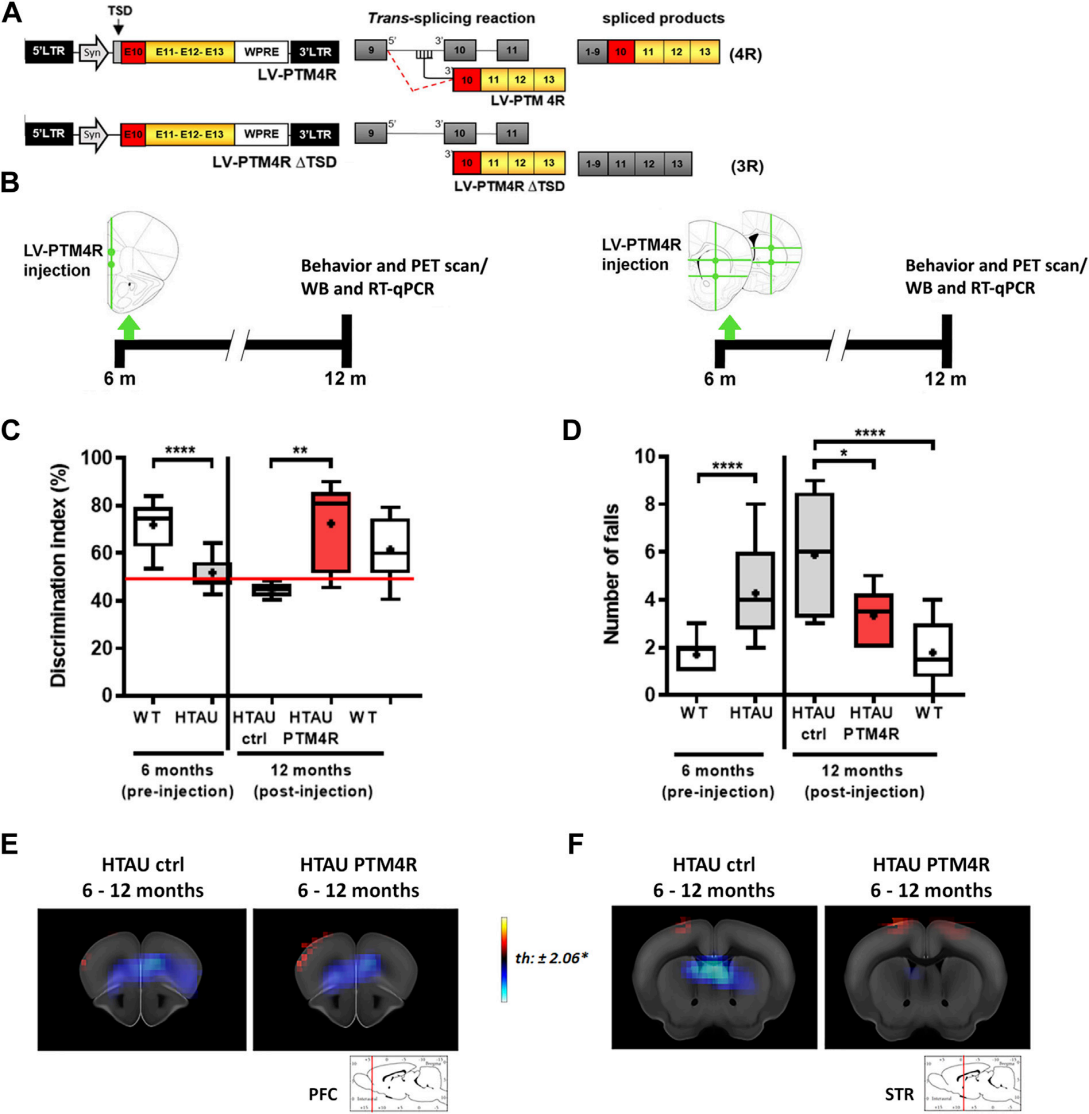
SMaRT rescues cognitive and motor deficits in htau mice. **(A)** Map of lentiviral vectors. LV-PTM4R used for trans-splicing and the expected chimeric transcript containing E10. Lentiviral vector LV-PTM4RΔTSD used for control mice. **(B)** Workflow of the experiments performed to modulate tau isoforms and evaluate phenotypic changes. Mice were bilaterally injected at 6 months old, either with PTM4R or control PTM4RDTSD into the prefrontal cortex at 2 ventral sites or into the striata at 2 anteroposterior sites, with 2 ventral sites each. **(C)** Novel object recognition task performed by htau mice before (6 months old, Unpaired t-test, ****p < 0.0001, WT *n* = 17, htau *n* = 21) and after (12 months old) PTM4R or control injection, compared to WT controls (1-way ANOVA, followed by Tukey’s multiple comparisons test, **p < 0.01, htau control *n* = 8, htau PTM4R *n* = 10, WT control *n* = 15). **(D)** Total number of falls in the rotarod before PTM injection (6 months-old; unpaired t-test, ****p < 0.0001, WT *n* = 23, htau *n* = 25) and post-injection (12 months-old; 1-way ANOVA, followed by Tukey’s multiple comparisons test, *p < 0.05, ****p < 0.0001, htau control *n* = 9, htau PTM4R *n* = 8, WT control *n* = 16) indicating that PTM4R htau mice showed a considerable reduction in falls, similar to WT, compared to their htau controls. **(E**,**F)** *In vivo* FDG-PET imaging of brain glucose uptake analysed at 12-month-old PTM4R or control htau mice compared to brain glucose uptake at 6 months old (before PTM injection). Red-yellow look up table side indicates [18F]-FDG uptake increases, while blue indicates uptake decrease. **(E)** PTM4R and control htau groups did not show significant difference in the mPFC brain glucose uptake (*n* = 8–10). **(F)** Striatal brain glucose uptake of PTM4R htau showed differences when compared to htau control (*n* = 8–9). T values for *p* < 0.05 are specified for each comparison. Images shown represent T-maps of brain metabolism compared to earlier recorded activity of the same groups.

**FIGURE 4:**
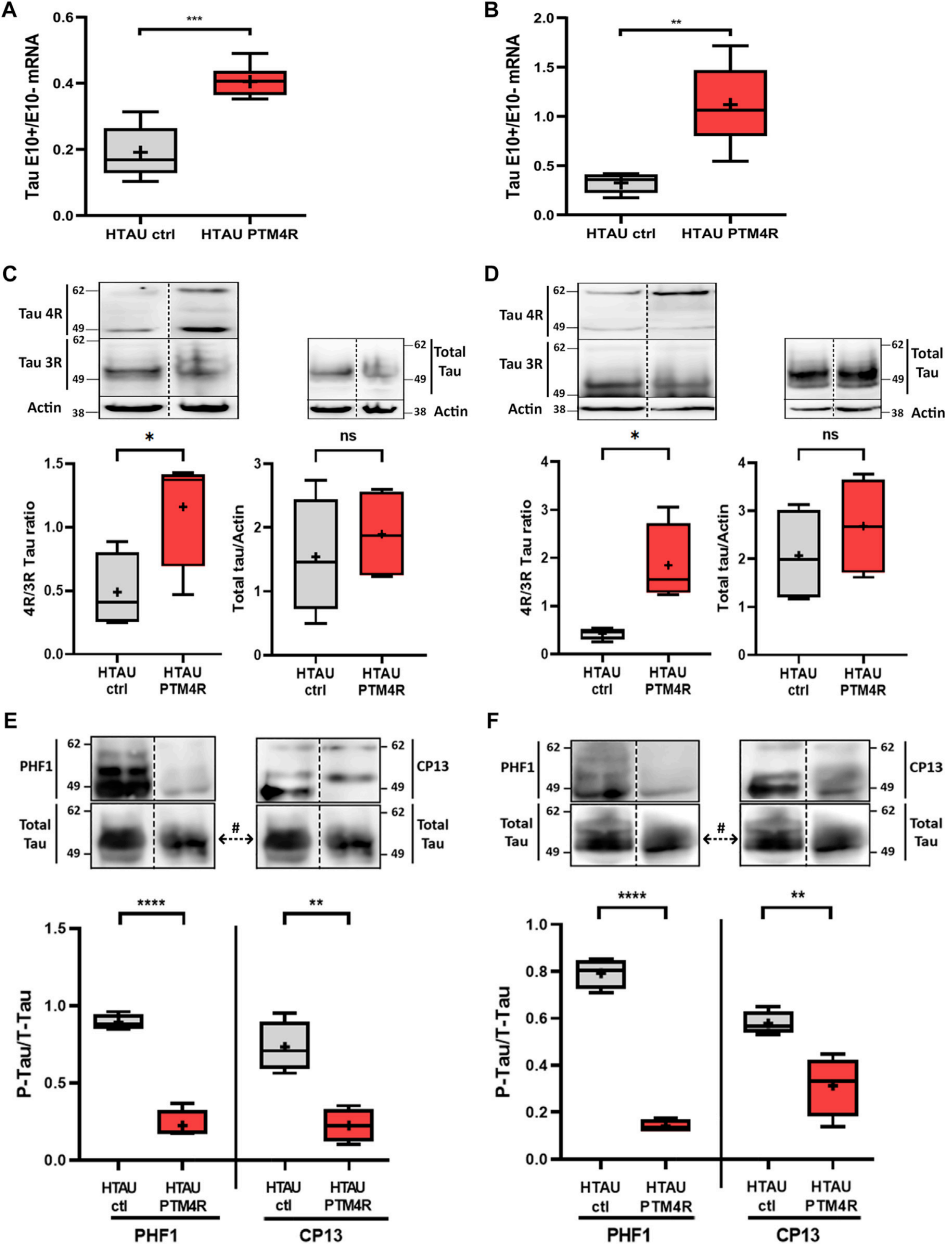
SMaRT rescue of Tau Isoforms and phosphotau contents in htau mice. **(A**,**B)** Relative content of tau isoforms determined by RT-qPCR with specific primers for 3R (E10-) and 4R (E10+). Data are expressed as the isoform ratio (4R/3R) from mPFC (unpaired t-test, ***p < 0.001, htau control *n* = 5, htau PTM4R *n* = 7) and striatum (unpaired t-test, p**<0.01, htau control *n* = 5, htau PTM4R *n* = 5). **(C**,**D)** Western blot detection of tau protein contents in homogenates with 3R and 4R in control and PTM4R groups, and the comparison between both in total tau protein for both structures: **(C)** PFC: 4R/3R ratio in htau ctrl and PTM4R groups (left) (Unpaired t-test, *p < 0.05, *n* = 4), and total tau (right) (no significant differences) **(D)** Striatum: 4R/3R ratio in htau ctrl and PTM4R groups (left) (unpaired t-test, *p < 0.05, *n* = 4) and total tau for both groups (right) (no significant differences). **(E**,**F)** Hyperphosphorylated tau protein contents determined by western blot in PFC **(E)** using PHF1(Unpaired t-test, ****p < 0.0001, htau control (*n* = 4) and htau-PTM4R (*n* = 4) and CP13 (Unpaired t-test, **p < 0.01, htau control (*n* = 4) and htau-PTM4R (*n* = 4)) or STR **(F)** PHF1(Unpaired *t*-test, ****p < 0.0001, htau control (*n* = 4) and htau-PTM4R (*n* = 4) and CP13 [Unpaired *t*-test, **p < 0.01, htau control (*n* = 4) and htau-PTM4R (*n* = 4)]. Values were related to Total Tau, # the same total tau blot was used to normalize phosphotau contents, detected with both (CP13 and PHF1) antibodies.

To determine if behavioural phenotypes observed in htau mice correlate with changes in brain metabolism in specific nuclei, we performed a microPET analysis with 18F-deoxyglucose (FDG-PET) to analyse glucose uptake in the whole brain of htau and WT mice while ageing. In the context of this study and based on our previous analyses of tau pathology in the htau model (Polydoro et al., 2009; Espíndola et al., 2018; Damianich et al., 2021), we focused on the analysis of brain nuclei directly related to the observed phenotypes, i. e the cortical areas and basal ganglia, that could be differentially affected in htau mice. Changes in 18F-FDG uptake were compared in the same cohorts of mice (WT and htau), at the following time slots: 3–6 months, 6–9 months and 9–12 months old. Both WT and htau mice showed increased glucose uptake in cortical areas at 3 months old compared to 6-month-old mice (Figures 2A,B; 3–6 months, WT and htau), consistent with more cortical activity in young mice (Brendel et al., 2017). However, in WT mice no changes were detected in 18F-FDG uptake after 6 months until 12 months old (Figure 2A, upper panel), while in htau, the mPFC (prelimbic and infralimbic areas) showed a decrease of glucose uptake from 6 to 9 months old, which was even more evident at 12 months old (Figure 2A, lower panel).

In turn, the striata of htau mice did not show significant changes in 18F-FDG uptake until 9 months old but display a drastic decrease between 9 and 12 months, while no changes in 18F-FDG uptake was observed in the striatum of WT mice over time (Figure 2B).

### 3.3 Phenotypic rescue by local tau isoforms modulation in htau mice

In previous reports (Espíndola et al., 2018; Damianich et al., 2021) we showed that local modulation of 3R:4R tau isoforms contents into the mPFC or the striatum of htau mice could *prevent* cognitive or motor coordination deficits, when such modulation was performed at 3 months old, before phenotypic onset. Hence, the most relevant question we aimed to address in this study was if these phenotypes could be *rescued*, once the deficits were already present.

Based on the FDG-PET imaging and the behavioural time-course studies performed, we chose to modulate tau isoforms at 6 months old, when both cognitive and motor phenotypes are present, and PET indicates initial signs of metabolic changes in brain nuclei.

We used the SMaRT *trans*-splicing strategy (Figure 3A) to drive E10 inclusion in the *MAPT* transcript to achieve equal tau 3R:4R contents, as previously described (Espíndola et al., 2018; Damianich et al., 2021). Htau mice were injected into the mPFC or the striatum with the PTM4R or controlPTM vectors at 6 months old and were analysed at 12 months old (Figure 3B). Before injection, 6 month old htau mice displayed no discrimination of novel object, showing an DI of 50% compared to ∼75% in WT mice (Figure 3C, left). At 12 months old, htau mice injected into the mPFC with control PTM showed similar or even worse deficit in the NOR task, while htau mice that had been injected with PTM4R displayed similar DI than age-matched WT controls (Figure 3C, right), indicating that PTM4R injection rescued cognitive decline of htau mice.

Similarly, htau mice injected in the striatum with PTM4R, improved their performance on the rotarod task (Figure 3D), as the total number of falls significantly decreased compared to their performance at 6 months old.

We next explored if PTM4R injection had an impact on glucose uptake in htau mice, as a readout for neurodegeneration progress. For this analysis we compared 12-month-old htau control and htau PTM4R mice versus 6-month-old htau mice. No significative difference in 18F-FDG uptake was observed in the mPFC between control and rescued mice, both PTM4R or control htau mice showed a similar reduction of glucose uptake between 6 and 12 months (Figure 3E), however the striata of htau control showed decreased in 18F-FDG uptake from 6 to 12 months old while this difference was not observed in htau PTM4R injected mice (Figure 3F). This result suggests a differential impact of tau isoforms shifting in the progress of neurodegeneration, between mPFC and striatum.

To validate the efficiency of the PTM4R injection we performed qPCR analyses to quantify the amount of *MAPT* transcripts containing or not the exon 10 (tau E10 + or tau E10-, respectively) to obtain a ratio between both isoforms. Either in the mPFC (Figure 4A) or in the striata (Figure 4B) of PTM4R injected htau mice, tau E10+/E10-isoform ratio was higher compared to htau controls. We next analysed 4R:3R tau protein isoforms contents by western blot in the mPFC and strita (Figures 4C,D) and verified that in both structures PTM4R injection led to a significant increase of 4R/3R ratio (>1), while control htau mice show over production of 3R Tau isoforms (4R/3R < 1), as previously reported (Andorfer et al., 2003; Espíndola et al., 2018). In addition, there was no difference in total tau between groups, indicating that PTM4R injection does not alter total tau contents. Total amounts of hyperphosphorylated tau were also measured in the PFC (Figure 4E) and Striatum (Figure 4F), using two different antibodies to detect phospho-tau at Ser202 (CP13) and Ser396/404 (PHF-1). A significant reduction of phospho-tau was observed in htau mice injected with PTM4R compared to htau control injected mice. This result indicated that PTM4R injection at 6 months prevent accumulation of phospho-tau in aged htau mice.

## 4 Discussion

In this study we found that local correction of abnormal exon 10 alternative splicing of the *MAPT* transcript could achieve a significant recovery of cognitive and motor impairments in a model of tauopathy. In the htau mouse brain the splicing processing of the human *MAPT* transcript favours the production of 3R tau in the adult brain. Thus, compared to the wild-type adult mouse brain that produces only 4R tau, the htau brain bears an aberrant content of tau isoforms. Former evidence indicates that such abnormal isoforms imbalance underlies cognitive and motor coordination impairments in aged htau mice (Duff et al., 2000; Andorfer et al., 2003; Espíndola et al., 2018). Indeed, our previous studies using SMaRT in this model showed that either cognitive or motor coordination impairments could be prevented by early modulation of *MAPT* E10 inclusion. Our challenge here was to determine if it was possible to rescue the phenotypes once they appeared, which would be more realistic in terms of translational approaches to the clinic.

To determine the time course of disease and the mainly affected brain nuclei, we assessed htau mice in behavioural tasks and microPET live imaging, using *in vivo* diagnosis like which is performed in human patients. Abnormal tau accumulation leads to a reduction in glucose uptake which is used as a marker of neurodegeneration in humans (Teune et al., 2010). In addition, several mouse models with tau pathology show changes in glucose metabolism (Bouter and Bouter, 2019; Endepols et al., 2022). In the htau mouse, abnormal overproduction of 3R tau leads to progressive phosphor-tau accumulation in specific brain nuclei (Andorfer et al., 2003; Polydoro et al., 2009; Espíndola et al., 2018). Therefore, we followed htau cohorts and their wildtype controls between 3 and 12 months of age, to determine whether htau mice display changes in glucose metabolism over time which could correlate with phenotypic changes and would serve to determine the most suitable window for therapeutic intervention. Between 3 and 6 months old, behavioural phenotypes became evident both in cognitive and motor coordination impairments. Imaging microPET analyses showed that htau mice did not show significant changes in glucose uptake until 9 months old. At this age the mPFC already showed a reduction in glucose uptake which worsens between 9 and 12 months old (Figure 2A). Yet, the striatum only showed a decrease in glucose uptake at 12 months-old, indicating a later imapriments in glucose metabolism in this structure. Together these results suggest that in the htau model, behavioural impairments precede changes in glucose uptake. These findings are consistent with the use of FDG-PET in the clinic, on which patients are usually evaluated after the onset of clinical signs of cognitive decline to determine affected nuclei to perform more accurate diagnosis (Ricci et al., 2020).

Based on the time course analyses, we performed SMaRT treatment at 6 months old, when behavioural phenotypes were already evident, but signs of neurodegeneration were not at advanced stages. This approach resembles a potential therapeutic intervention at early stages of tauopathy in human patients.

Our results showed that both cognitive and motor coordination impairments were rescued when E10 splicing modulation was performed either at the mPFC or striatum, respectively. The behavioural phenotypic rescue observed after local modulation of tau isoforms imbalance could be related, either to the significant reduction in the accumulation of pathological tau species (i.e., reduction of toxic tau species), and/or to the presence of functional 4R tau, yielding a recovery of *loss of function* in the htau mice. It is evident that in both structures PTM4R increased the 4R/3R ratio, without affecting total tau contents (Figures 4A–D). Moreover, phosphotau contents were reduced in both nuclei after PTM4R injection, as a readout of tau pathology reduction.

Noteworthy, FDG-PET analyses of htau mice at 12 months old did not show a major difference of glucose uptake reduction in the mPFC of PTM4R-htau mice compared to 6 month-old htau mice (Figure 3E) while the striatal injection of PTM4R seems to preclude the progress of metabolic changes, as there was no glucose uptake reduction (FDG PET similar glucose uptake between 6 and 12 months old in PTM4R htau mice; Figure 3F). This result may correlate with the differential progression of neurodegeneration observed by FDG PET between mPFC and the striatum (Figure 2). At the time of PTM4R injection (6 months-old) the striatum does not show metabolic changes, therefore, the PTM4R injection could have precluded these changes to take place in aged htau mice. In turn, the mPFC already shows FDG-uptake changes at 6 months old and although PTM4R could have reduced the increase of metabolic changes, those which were already established were not reverted. Indeed, after PTM4R injection we still observed a reduction in glucose metabolism in the htau PFC between 6 and 12 months, similar than in htau control mice (maybe because many neurons undergone degeneration before treatment, Figure 3E). Nevertheless, the observed functional recovery in the NOR task suggests that the remaining neurons of the PFC can recover function and rescue behavioural function upon 4R tau increase. Also, the lack of FDG-uptake changes in the PFC upon PTM4R injection might also indicate that the neurodegenerative process is still ongoing. On the other hand, as the striatum does not evidence changes in glucose metabolism at the time of injection -6 months old-, the neurodegenerative process might not have started yet. PTM4R injection at 6 months might therefore prevent such metabolic changes to occur, precluding the FDG-PET changes. It would be necessary to perform long term analyses to further determine whether modulation of tau isoforms could stop or delay the neurodegenerative process, and if the outcome depends on the treated brain area. Indeed, such differences between mPFC and striatum are likely to rely on neuronal selective vulnerability. Excitatory neurons of cortical areas are known to be more vulnerable to pathological tau accumulation than GABAergic neurons (Fu et al., 2018; Leng et al., 2021). Therefore, selective vulnerability might affect not only the time course of the neurodegenerative process in each neuronal subpopulation, but also the outcome of treatment with disease-modifying therapies. This is an important point that needs to be considered to further determine the most accurate therapeutic interventions in tauopathies that affect different brain nuclei and neuronal subpopulations.

In summary, results presented in this study are a proof of concept that local regulation of abnormal *MAPT* splicing could be an effective approach to stop the progression of tauopathy.

RNA-based therapies have many advantages over classic gene therapy/gene-edition approaches and recently arose as promising tools to treat human diseases (Drew, 2019; Dammes and Peer, 2020). Particularly, the correction of abnormal E10 splicing by RNA reprogramming has been tested using several methods, such as antisense oligonucleotides (ASO) (Schoch et al., 2016; Suñé-Pou et al., 2017) (already in Phase 1b clinical trial) or morpholino exon skipping (Sud et al., 2014). In this scenario, SMaRT could provide an alternative, versatile therapeutic strategy which will allow not only modulation of either 3R or 4R imbalances but also the correction of mutations downstream E10 in the *MAPT* transcript (Rodriguez-Martin et al., 2009). Hence, RNA-therapy approaches based on the SMaRT strategy for tau RNA reprogramming represent a promising tool to be explored in translational studies.

## Data Availability

The original contributions presented in the study are included in the article/supplementary material, further inquiries can be directed to the corresponding author.
